# Evaluation of gastric acid in dependence of pre-operative fasting

**DOI:** 10.1186/2197-425X-3-S1-A741

**Published:** 2015-10-01

**Authors:** BA Tudor, GA Roth, WD Huber, O Kimberger, CG Krenn, L Triffterer

**Affiliations:** Medical University of Vienna, Department of Anesthesiology, General Intensive Care and Pain Medicine, Vienna, Austria; Medical University of Vienna, Department of Pediatrics and Adolescent Medicine, Vienna, Austria

## Introduction

It is generally accepted that fasting reduces the risk of regurgitation and aspiration of gastric contents during induction of anaesthesia and surgery. Children, like adults, are required to fast before general anaesthesia to reduce volume and acidity of their stomach contents.[1] Fasting while waiting for surgery can be a stressful event for the children and their parents. To be removed from their daily routine and potential fear of the procedure, sometimes also aggravated by their nervous parents, can cause anxiety as well as uncooperative behaviour, which might endanger the safe performances of the anesthesiological and surgical procedures. To prevent long-lasting fasting 'Nutricia preOP®' - a carbohydrate containing drink - was introduced.[2]

## Objectives

The first aim of the study is to demonstrate the safety of consumption of clear fluids up to two hours preoperatively and their impact on gastric volume and pH.

The second aim is to study a possible difference in the incidence of postoperative nausea and vomiting. Further we evaluate the influence of preOP^®^ on hunger, thirst, tiredness and weakness, in comparison with normal fasting. As primary outcome we assume not significant difference properties between preOP®-Group versus the conservative fasting group.

## Methods

We evaluate the difference in the gastric volume and pH in 40 children - between 2 and 18 years- who undergo a routine gastroscopy in general anaesthesia. The first group of 20 patients adhere to the regular preoperative fasting period of 6 hours or more. The second group of 20 patients is allowed to consume 5 ml/kgBW of preOP^®^ in the evening and up to two hours before the procedure. The gastric content was collected during the planned routine gastroscopy and afterwards the pH and the volume were measured. The patients were asked about their sensation of nausea and vomiting, their pain-level, hunger, thirst, tiredness and weakness before entering and when leaving the anaesthetic recovery room and 1 day postoperative using the KUS-scale by Büttner and the Face-pain-scale by Wong-Baker.

## Results

Obtained results of patients drinking preOP^®^ show a mean volume of gastric content of 8 ml (p < 0,05) and a pH of 2 (p < 0,05). In the control group a mean gastric volume of 13 ml (p < 0,05) and a pH of 1 (p < 0,05) could be analysed. Furthermore the drink shows a subjective well-being improvement in influence of hunger and thirst but no differences in tiredness and weakness, in comparison with normal fasting.

## Conclusions

This study demonstrates the safety of consumption of preOP^®^ up to two hours preoperatively. Especially for very small children a shorter fasting time helps decreasing potential fear of the surgery and so it might increase patients' compliance for the necessary preoperative procedures.
